# Tracing the Neovolcanic zone along the sediment-covered regions of the Red Sea Rift

**DOI:** 10.1038/s41598-025-89332-2

**Published:** 2025-02-14

**Authors:** Jonas Preine, Nico Augustin, Neil C. Mitchell, Froukje M. van der Zwan, Annne-C Wölfl, Martin Schade, Yousif A. Izzeldin, Mudather A. M. Osman, Christian Hübscher

**Affiliations:** 1 Institute of Geophysics, University of Hamburg, Bundesstrasse 55, 20146 Hamburg, Germany; 2 Department of Geology and Geophysics, Woods Hole Oceanographic Institution, Woods Hole, MA 02543 USA; 3 GEOMAR Helmholtz Centre for Ocean Research Kiel, Wischofstraße 1-3, 24148 Kiel, Germany; 4 Department of Earth and Environmental Sciences, University of Manchester, Williamson Building, Oxford Road, Machester, M13 9PL UK; 5 King Abdullah University for Science and Technology KAUST, Thuwal, Saudi Arabia; 6 University of Kiel, Kiel, Germany; 7 Awasconrc, Gereif W, H4, Bld 376, POB 410, Khartoum, Sudan; 8 Institute of Marine Research, Red Sea University, Port Sudan, Sudan

**Keywords:** Red Sea, Submarine volcanism, Reflection seismic, Mid-ocean ridges, Bathymetry, Geodynamics, Geophysics, Volcanology

## Abstract

The Red Sea Rift is an ultra-slow spreading rift filled with Miocene salt and younger sediments. While volcanic features can be observed in exposed areas in the southern Red Sea Rift, evidence of volcanism in the sediment-blanketed regions in the central and northern Red Sea Rift has been lacking, leaving open whether the mid-ocean rift axis continues beneath them. Here, we present new multichannel seismic and high-resolution bathymetric data of these blanketed regions. Our data reveals multiple instances where oceanic crust can be traced beneath the evaporite cover, forming volcanic edifices protruding through the sediment cover. We identify abundant circular depressions in the sediment cover as volcanic craters, which formed by deep-sea explosive volcanism or caldera collapses. The common occurrence of volcanic features in the sediment-covered regions supports the continuous formation of oceanic crust along large parts of the Red Sea Rift.

## Introduction

**Fig. 1:**
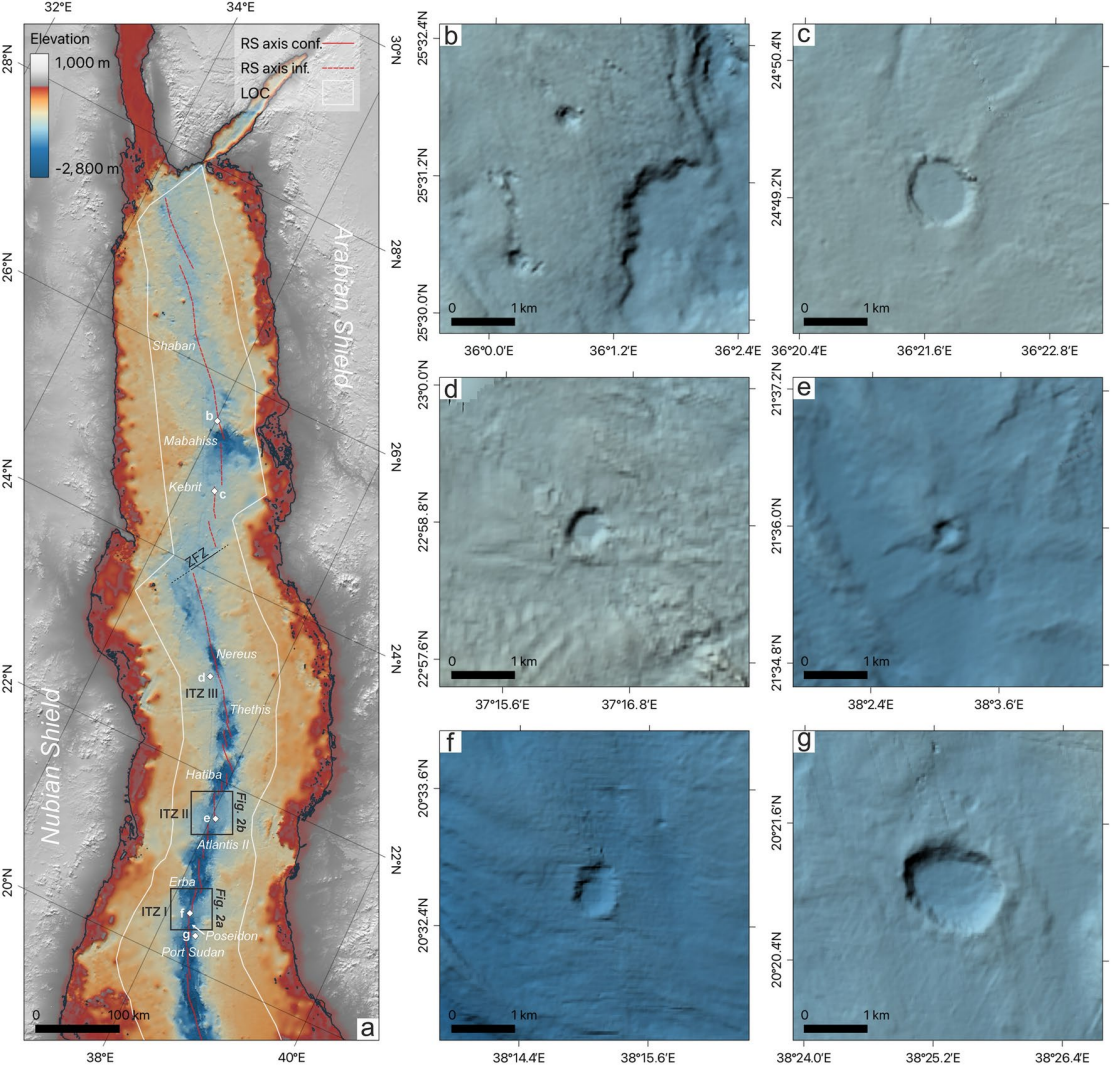
Morphological maps of the Red Sea with crater structures. (**a**) GEBCO 2020 bathymetry of the central and northern Red Sea (resolution of 435 m at 20°N). Bathymetric depressions within exposed oceanic crust (“Deeps”) are labelled in italicized text. Notable features include the Inter Trough Zone (ITZ), and the interpreted Zabargad Fracture Zone (ZFZ), Limit of Oceanic Crust (LOC), and the confirmed (RS axis conf) and inferred (RS axis inf) axes of the Red Sea (after Augustin et al.^4^). Letters refer to (**b**–**g**), boxes indicate the areas shown in Fig. 2. (**b**–**g**) Crater structures observed in the sediment-covered parts of the Red Sea Rift. Maps were generated using QGIS (version 3.34, https://qgis.org).

The Red Sea is a narrow ocean basin between the Neoproterozoic Nubian (African) and Arabian shields^1^ (Fig. 1a). Large parts of the Red Sea Rift are buried under thick blankets of Miocene salt and sediments^2–6^. As a result, the rift axis and underlying crust are hidden from direct observations in the sediment-filled Inter-Trough Zones (ITZ) in the central Red Sea as well as in the northern Red Sea north of 23.3°N^3–5^ (Fig. 1). Hence, the nature of the crust in the Red Sea, the distribution of oceanic crust under the sediment cover, and the exact position of the rift axis are still under discussion^5,6^.

In general, two endmember-models are discussed for the Red Sea Rift:

- Nodes of seafloor spreading grow and develop a mature rift southward; no oceanic crust is present in the ITZs and in the northern Red Sea, and oceanic crust is not older than 5 Ma^7–9^. The ITZs are underlain by continental crust.
- Continuous spreading occurred since 8–14 Ma^5,6,10^. The continuous spreading rift stretches continuously from the southern to the northern Red Sea. Oceanic crust underlies the evaporites along the entire Red Sea and crops out (1) where basement obstacles inhibit the salt flows from filling the rift valley; (2) where plate-tectonic separation rate has exceeded the rate of salt flowage^5,6,11^.

**Fig. 2:**
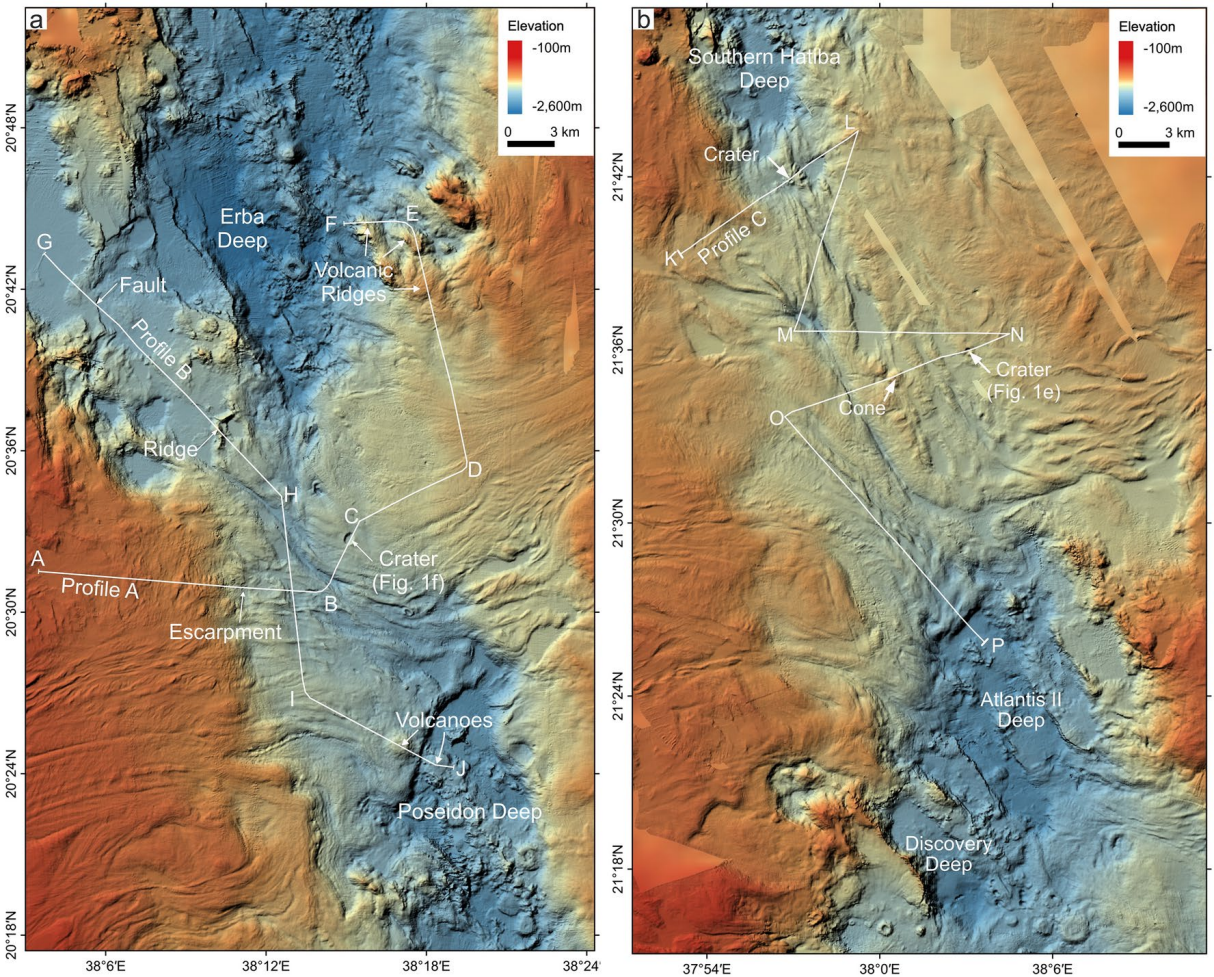
Morphological maps of the northern and southern Inter-Trough Zones. Morphological map of Inter-Trough Zones (ITZs) with seismic profile layout from Expedition 64PE-445 (Augustin et al.^30^). Shown are the southern ITZ I (**a**) and the northern ITZ II (**b**). Prominent morphological structures, such as craters and cones are marked. Knickpoints of seismic profiles are marked with letters. Maps were generated using QGIS (version 3.34, https://qgis.org).

Regardless of the nature of the Red Sea crust, full spreading rates are at the ultra-slow end for mid-ocean ridges, varying from < 10 mm/yr north of 25°N to about 16 mm/yr at around 18°N^12–14^, consistent with the morphology of the exposed mid-ocean ridge crust^4^ and gabbro fragments found in the Discovery Deep, which is typical for ultra-slow ridges^15^ (Fig. 2b). Also, both models agree that full oceanic spreading occurs in the southern Red Sea, where oceanic crust can directly be observed and sampled. From the northern Red Sea, north of 23.3°N, samples of mid-ocean ridge basalts and, thus, direct sampling of oceanic crust are only reported from the Mabahiss Deep area (25°N) and Shaban Deep (26.2°N)^16,17^ (Fig. 1a). In the central Red Sea, from Nereus Deep southwards, mid-ocean ridge basalt is present in more of these windows, but in-between the basement is covered by the Miocene salts in ITZs. In these ITZs and sediment-covered regions, no direct observations of volcanoes were reported.

However, within the central Red Sea, crater-like structures have been observed in high-resolution bathymetry lying within the ITZs and under salt and sediment flows^4^ (Fig. 1b–g). Based on their morphology, particularly their elevation rims, and their location within the rift, they have been interpreted to be of volcanic origin^4^. Yet, so far, the connection of these surface features to the sub-seafloor structure has remained unconstrained since, for most parts of the Red Sea, the coverage or accessibility with reflection seismic profiles is sparse, and the quality of available seismic lines is generally poor. In particular, seismic profiles across the ITZs of modern quality are largely missing in the literature.

Typically, seismic reflection profiles in the Red Sea show an upper unit containing Pliocene–Pleistocene (PP) sediments and a lower unit containing Miocene evaporites^18^. The PP sediments generally correspond to the upper 200–300 ms TWT (approx. 230–350 m) and consist of hemipelagic deposits^2,8,19–21^. Away from the coast, the lower hemipelagic PP sediments are commonly transparent^22,24^. Below these sediments lies a high-amplitude, low-frequency reflection that was termed the S-reflection^20,25^. Correlation with DSDP Sites 225, 227, and 228 shows that the S-reflection aligns with the top of the Miocene evaporites or a rigid shale immediately above them^26^. The surface of the S-reflection has been mapped throughout many flank areas of the Red Sea and lies generally sub-parallel to the seafloor^26^. Towards the coasts, evaporites reach thicknesses of several kilometres^10^, and extensive diapirism occurs in the shelf regions, gradually becoming less pronounced towards deep waters^27^. Here, the evaporites are thought to be more in the “pillow” stage and are affected by flowage towards the spreading axis, effectively thinning out^3,27^.

The high amplitudes of the S-reflection are caused by an extreme impedance contrast, where the p-wave velocity changes from 1.9 km/s in the PP sediments to 4.2 km/s within the evaporites^26,27^. This high-impedance contrast considerably limits the seismic signal penetration^20^. Therefore, seismic images showing the base of the evaporites are extremely sparse and depict only interrupted to highly scattered reflections, which are especially complex along the ITZs^10,28,29^.

This paper presents new multichannel seismic and high-resolution bathymetry data to investigate the subsurface structure of the ITZs in the central and northern Red Sea. We show two seismic profiles crossing the southern ITZ I (Fig. 2a) and one profile crossing the northern ITZ II (Fig. 2b). Our objectives are to (1) image the base of the sediment and evaporite cover in these areas, (2) investigate their relation to the exposed oceanic crust outside the ITZs, and (3) unravel the roots and nature of the crater- and cone-shaped structures visible at the seafloor. Ultimately, this study aims to provide further evidence to solve the debate regarding the presence of oceanic crust underneath the ITZs and the continuation of the spreading axis along the Red Sea Rift.

## Results

### Seismic reflection profiles across inter-trough zones

**Fig. 3:**
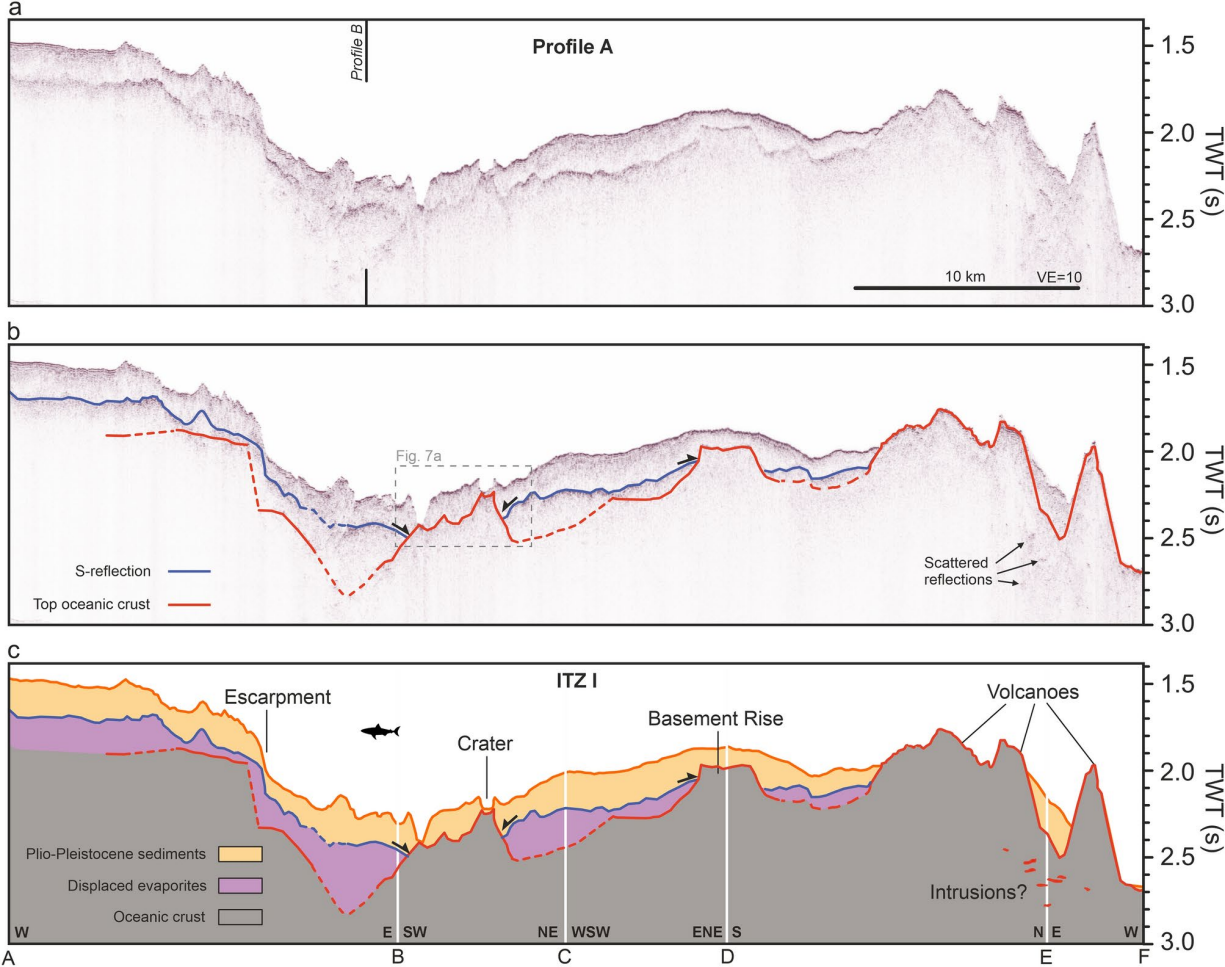
Seismic profile A crossing ITZ I west to east. (**a**) Uninterpreted seismic profile. Black vertical line indicates crossing location of Profile B (Fig. 4). VE: Vertical Exaggeration (estimated using 1500 m/s). (**b**) Seismic profile with prominent reflections marked. Blue marks the S-reflection, red the top of the oceanic crust. Dashed lines indicate interpreted horizons with lower confidence. Black arrows indicate onlap terminations. The dashed box outlines the detailed view in Fig. 7a. (**c**) Interpreted seismic profile. Units are manually interpolated through areas without distinctive reflections. For location, see Fig. 2.

Profile A crosses the southern ITZ (ITZ I) from west to northeast across two glacier-like salt flow structures that meet in the centre of the ITZ (Figs. 2, 3). Starting at the western side of ITZ I, the topography decreases gently before reaching a major step visible in the bathymetry (Fig. 3a). Afterwards, the profile crosses the deep, central part of the ITZ (Fig. 3a). From here, it continues towards the northeast and traverses the crater-like structure visible in the bathymetry (between markings ‘B’ and ‘C’; Fig. 3a, 1f). The profile continues along the eastern glacier-like salt flow structure and ends crossing two volcanic ridges at the northern margin of ITZ I (Fig. 3a).

Throughout most parts of the profile, we identify a high-amplitude reflection at approximately 200 ms TWT below the seafloor, separating chaotic to slightly laminated strata from an incoherent, transparent basement (marked blue in Fig. 3b). This high-amplitude reflection lies approx. sub-parallel to the seafloor in some parts, e.g., in the western part of the profile between markers A and B, Fig. 3b). In other parts, we observe differences in the thickness and rough topography.

East of the bathymetric escarpment (Fig. 3), the seafloor has an irregular topography, and the S-reflection becomes harder to trace, appearing scattered or losing visibility (Fig. 3b). East of the crater, the S-reflection is visible again as a distinct reflection. It is interrupted by an angular reflection (marked ‘basement rise’, Fig. 3c) and loses visibility towards the northern margin of ITZ I, where the profile crosses major volcanic ridges (Fig. 3c).

In the central part of ITZ I, we identify a deeper reflection rising towards the east (marked red in Fig. 3b). It crops out at the deepest point of the ITZ, where it is onlapped by the S-reflection (Fig. 3b). Towards the east it rises further and reaches the surface of the crater (Fig. 3b, c). Northeast of the crater, it dips downwards and is then again onlapped by the S-reflection before losing visibility further towards the east (Fig. 3a). Faint signatures of a reflection underneath the S-reflection (marked dashed red, Fig. 3b) can be seen rising towards the basement rise and towards the volcanic ridges in the north, connecting to the seafloor (Fig. 3b). Underneath these ridges, we observe scattered reflections. Outside the ITZ, we identify no reflection underneath the seafloor (Fig. 3b).

**Fig. 4:**
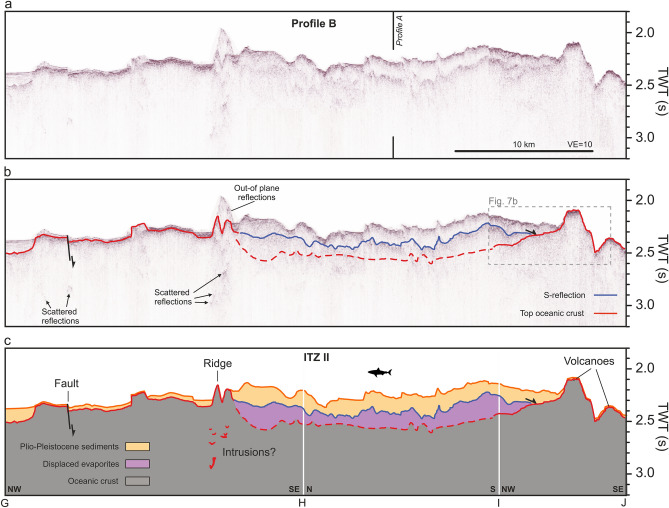
Seismic profile B crossing the southern ITZ north to south. (**a**) Uninterpreted seismic profile. Black vertical line indicates crossing location of Profile B (Fig. 3). VE: Vertical Exaggeration (estimated using 1500 m/s). (**b**) Seismic profile with prominent reflections marked. Blue marks the S-reflection, red the top of the oceanic crust. Dashed lines indicate interpreted horizons with lower confidence. Black arrows indicate onlap terminations. The dashed box outlines the detailed view in Fig. 7b. (**c**) Interpreted seismic profile. Units are manually interpolated through areas without distinctive reflections. For location, see Fig. 2.

Profile B crosses ITZ I from the northwest to the southeast (Figs. 2, 4). The rift axis north of ITZ 1 shows a flat topography that is cut by several distinct faults that are also clearly visible in the bathymetry (Figs. 2, 4). A thin sediment cover is underlain by a high-amplitude reflection that crops out at a prominent ridge marked in Fig. 4c. Here, the seafloor reflection is scattered as a result of the complex ridge topography, which induces out-of-plane reflections and in-plane scattering above the seafloor (Fig. 4). Scattered reflections are visible underneath the ridge. Further east, in the ITZ, we identify the S-reflection, which roughly follows the shape of the seafloor at a depth of about 200 ms TWT (Fig. 4a, b). We can correlate this reflection to the S-reflection in Profile A (Fig. 3) at the intersection of both profiles and trace this reflection towards the southern margin of ITZ I, where it onlaps a volcanic edifice (between markings ‘I’ and ‘J’, Fig. 4c). Here we identify a deeper reflection that rises from the north-west forming the flanks of the volcano and cropping out at the seafloor (Fig. 4). Towards the south-east, this reflection forms the seabed (Fig. 4).

**Fig. 5:**
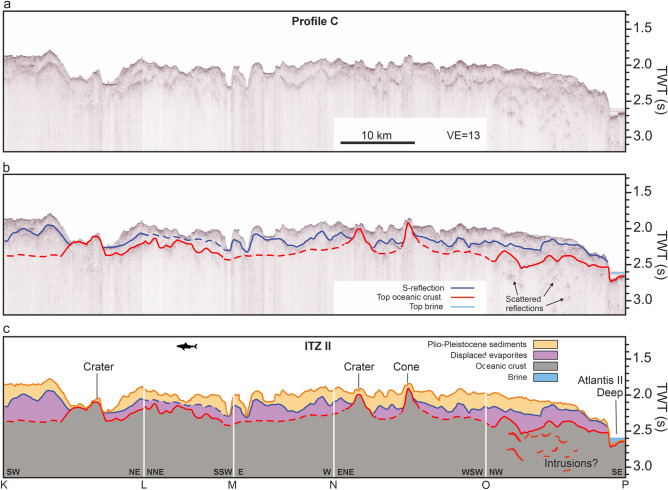
Seismic profile C crossing the northern ITZ. (**a**) Uninterpreted seismic profile. VE: Vertical Exaggeration (estimated using 1500 m/s). (**b**) Seismic profile with prominent reflections marked. Blue marks the S-reflection, red the top of the oceanic crust. Dashed lines indicate interpreted horizons with lower confidence; (**c**) Interpreted seismic profile. Units are manually interpolated through areas without distinctive reflections.

Profile C crosses the northern ITZ (ITZ II) from north to south, crossing Atlantis II Deep (Figs. 2, 5). The profile zigzags across the centre of ITZ II, crossing several prominent structures in the bathymetry labeled ‘Crater’ or ‘Cone’ (Figs. 2, 5). The seafloor undulates over a complex subsurface structure (Fig. 5). Similar to Profiles A and B, we identify the S-reflection as a distinct high-amplitude reflection, following the seafloor at a depth of about 200 ms TWT, but being repeatedly interrupted (Fig. 5b). The S-reflector is most clearly recognizable in the central part of the profile between markers M and N, although interruptions can also be observed here (Fig. 5b). Repeatedly, the faint signature of a deeper reflection can be recognized, which rises to the seafloor at several locations, for example where it is marked as ‘Crater’ and ‘Cone’ (Fig. 5c). Between markers O and P, we observe scattered reflections of complex geometries (Fig. 5b). The Atlantis II Deep in the south forms the lowest point of the profile (Fig. 5). Here we recognize a perfectly straight reflection above the sea floor, which is typical for the surface of the brines in the Red Sea^21^ (Fig. 5a). The seafloor reveals a high-amplitude reflection with no deeper reflection observable (Fig. 5a).

### Bathymetry along a crater structure in the kebrit deep

**Fig. 6:**
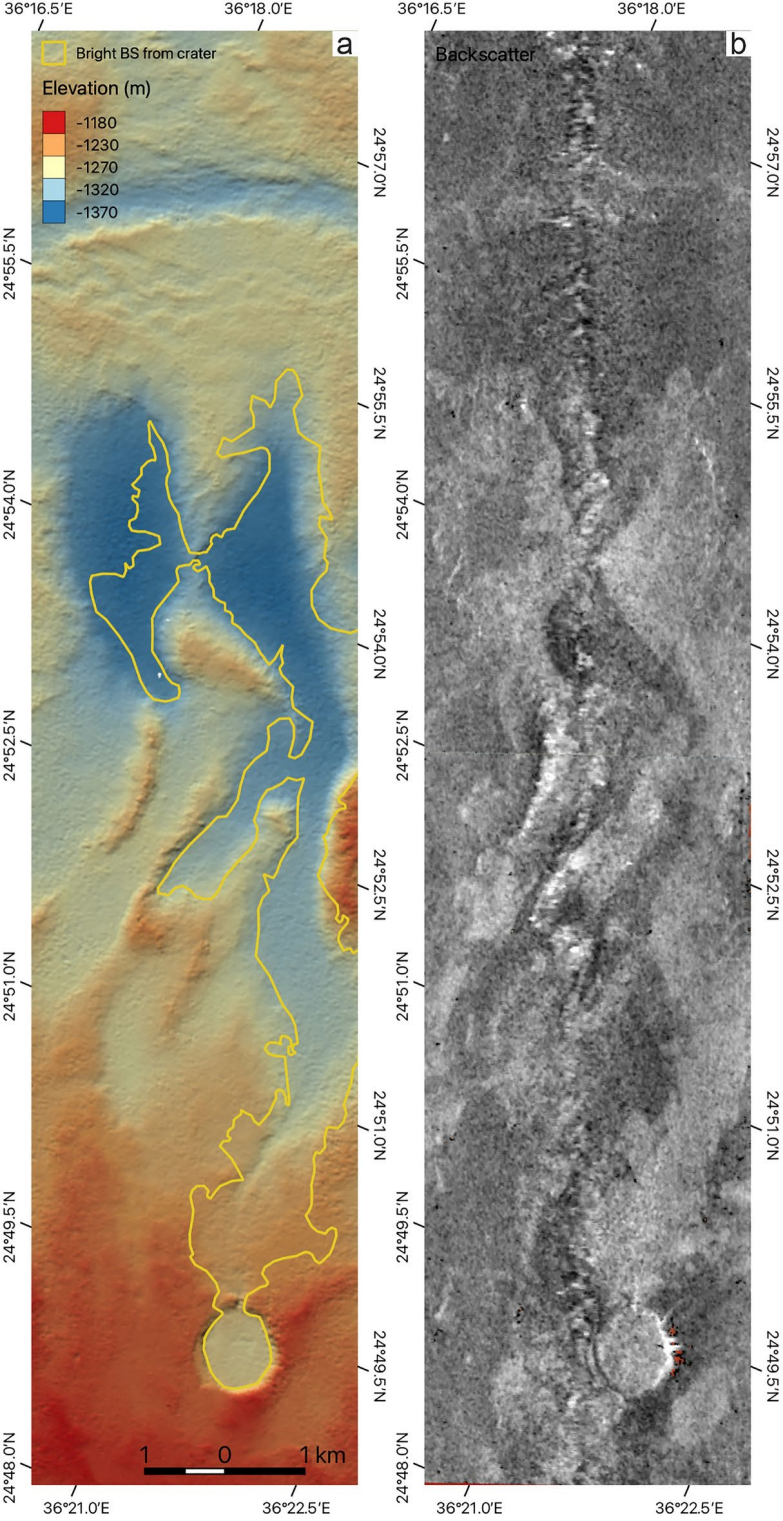
Morphological map of a crater structure with a recent lava flow. (**a**) Bathymetric map and (**b**) backscatter map of a crater structure close to Kebrit Deep plotted with positive polarity (high backscattering represented by lighter tones)^29^. The yellow lines in (**a**) indicate the interpreted outline of a lava flow associated with the crater according to areas of high backscattering. BS: backscatter. Maps were generated using QGIS (version 3.34, https://qgis.org).

Figure 6 presents a detailed high-resolution bathymetric map and an accompanying backscatter map of the Kebrit Deep, situated in the northern Red Sea, based on publicly available data^29^ (Fig. 1). In the southern portion of the mapped area, we identify a prominent circular, crater-like structure with a diameter of approximately 1 km (Fig. 6a). This feature is characterized by elevated rims, which are interrupted to the north (Fig. 6a). The floor of the crater exhibits distinctly high backscatter values and is connected to an elongated, channel-like area of high backscatter that extends for roughly 6 km in a northward direction (Fig. 6b). This elongated feature is constrained within a channel-like confinement and appears to connect to areas of lower bathymetry in the northern region. The crater itself is positioned on an elevated section of the seafloor (Fig. 6a, b). Further to the north, the channel-like area of high backscatter transitions into regions of bathymetric lows and is periodically interrupted by ridge-like structures (Fig. 6a, b).

## Discussion

Our seismic profiles reveal that the ITZs predominantly exhibit a typical Red Sea subsurface structure: a transparent to slightly stratified PP unit approximately 100–200 ms TWT thick, underlain by a pronounced reflection, the S-reflection (Figs. 3, 4, 5). Both, evaporites and PP sediments lose internal layering towards the rift axis^30^ (Figs. 3, 4, 5). Beneath this, the seismic images show an incoherent acoustic basement (Figs. 3, 4, 5). In several locations, we identify a third coherent reflection angular to the seafloor and the S-reflection (marked red in Figs. 3, 4, 5). The occurrences of this third reflection are associated with either (1) the surface of the deeps at the margins of the ITZs (Figs. 3, 4, 5) or (2) outcropping volcanic structures within the ITZs (Figs. 3, 4, 5).

**Fig. 7:**
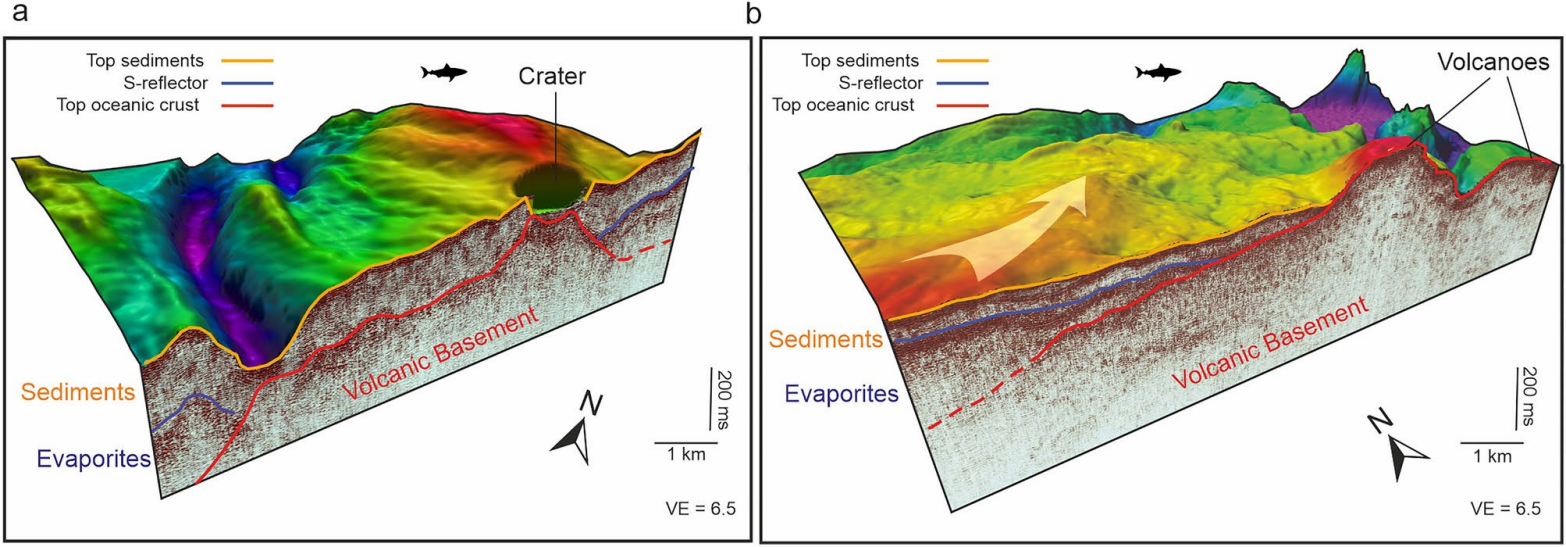
3D views of the seismic profiles combined with bathymetry of volcanic structure within the southern ITZ. (**A**) Crater-like structure, where the volcanic basement rises towards the surface. Towards the NE, this reflection is onlapped by the S-reflection. For location, see Fig. 3. (**B**) Volcanic edifice visible cropping out in the southern ITZ. The seismic profile shows a high-amplitude reflection rising toward the surface and forming the top of the volcanic edifice. It is onlapped from the southwest by the S-reflection. The flow direction of the salt is indicated by an arrow, showing a deflection next to the volcanic edifice. For location, see Fig. 4. The 3D model was created using Fledermaus software (version 7.7, https://www.qps.nl/fledermaus) and seismic data extracted using Kingdom Seismic and Geological Interpretation Software (version 2019, https://www.software.slb.com/products/kingdom).

The most prominent example of a third reflection connecting to outcropping volcanic structures is the crater in Profile A, where an ascending reflection can be traced over several kilometres below the S-reflection, which rises to the base of the crater (Fig. 3). This reflection is onlapped by the S-reflection from the west and the east (Fig. 3). Figure 7a highlights this part of the profile, providing a three-dimensional view of the seismic profile together with the bathymetry. The crater structure is visible as a distinct depression in the seafloor and the red reflection rises to the base of this crater (Fig. 7a). Another prominent instance of a third reflection below the S-reflection ascending to form the surface of a seabed volcano, can be observed in Profile B (Fig. 4). Figure 7b highlights the spatial relation of the third reflection, the volcano, and the S-reflection in this area in detail. It shows that the volcano acts as a buttress deflecting the direction of the glacier-like flow of the evaporites (see arrow in Fig. 7b), implying a direct control of the volcanic basement on the direction of a salt flow^4^ (Fig. 7b). Also, at the northern ITZ II in Profile C, we identify examples where seabed volcanic structures like craters and cones correlate with the emergence of a faint third reflection below the S-reflection (red horizon in Fig. 5b).

The spatial correspondence of a third reflection and outcropping volcanic structures suggests that it represents the volcanic basement of the oceanic crust that continues underneath the ITZs as suggested by Augustin et al.^5,31^. Where the evaporites exceed a thickness of about 150–200 ms TWT (~ 300 to 400 m), the volcanic basement loses visibility (e.g., Fig. 4). This can be explained as the result of the comparably small acoustic energy emitted by the Sparker system (6 kJ), the relatively short streamer length (100 m) and the extreme acoustic impedance contrast at the sediment-evaporite interface^32^. Even in rare examples of seismic data from vintage long-offset surveys with G guns, the acoustic basement underneath the evaporites is hardly visible and appears only as faint, scattered reflections^10,30^.

Vintage seismic profiles crossing the northern and southern ITZ have been presented by Izzeldin^10^ showing a strong reflection sub-parallel to the seafloor, identified as the S-reflection. Similar as observed here, below the S-reflection, there are diffuse reflections that rise to the surface at the margins of the ITZs towards the Deeps^10^. Also, Izzeldin^10^ interpreted these scattered reflections as oceanic crust that continues along the entire length of ITZ I and ITZ II. Another set of long-offset seismic profiles has been presented by Mitchell et al.^29^. Profile 50 crosses the western shoulder of the Red Sea Rift west to east and ends at the northern edge of ITZ I (Mitchell et al.^32^). It shows a diffuse, but continuous reflection that can be traced underneath ITZ I. Mitchell et al.^32^ interpret that this reflection corresponds to the oceanic basement. In both cases, the depth and characteristics of the reflections interpreted as oceanic crust correspond to the reflections in the here-studied Sparker profiles that connect to the exposed oceanic crust. Taking these interpretations and our observation of the spatial correspondence of a third reflection and volcanic edifices together, we have strong evidence for a continuation of oceanic crust underneath the ITZs with widespread volcanism that is mostly covered, but several edifices stick out of the evaporite/sediment cover. Thus, our study supports the endmember model B for the Red Sea Rift in the central Red Sea: a continuous spreading of oceanic crust^5,6,11^, challenging the model of separated nodes of oceanic basins^7,9^.

Our study implies that the crater crossed by Profile A within the southern ITZ is correlated with a rise of the oceanic crust (Figs. 3, 7a). Similarly, crater structures in Profile C crossing the northern ITZs correlate with an ascending volcanic basement, indicating a volcanic origin (Fig. 5). Augustin et al.^4^ discussed the potential origin of these crater structures and concluded that a formation by phreatic or phreatomagmatic eruptions is most likely, based on the morphology and ejecta rim around the craters. The presence of a third reflection, interpreted as oceanic crust, rising to the seafloor underneath these structures strongly supports this interpretation (Figs. 3, 4, 5, 7).

A volcanic origin of the observed craters is further indicated by similar craters in the northern Red Sea, particularly, the crater structure to the Kebrit Deep (Figs. 1, 6). The high backscattering and the flow-like morphology lead us to the interpretation of a lesser sedimented, likely Holocene (cf. Augustin et al.^4^) lava flow lying north of the crater at the seafloor (Fig. 6). As the origin of this flow starts in the crater, it supports the crater as a volcanic feature which had a crater-forming eruption followed by effusive lava flow expulsion. From these instances that confirm a volcanic origin as the most likely explanation for the creation of the crater structures, we can infer the continuous presence of oceanic crust along the sediment-covered regions of the Red Sea Rift, up to at least Mabahiss Deep in the northern Red Sea (Fig. 1).

Profiles A-C show areas, where deeper scattered reflections are visible (Figs. 3, 4, 5). These have complex geometries and occur underneath what we interpret as the top of the oceanic crust. Similar reflections have been noted in Discovery Deep, where a prominent concave reflection has been noted underneath the oceanic crust, interpreted as sill reflections^15^. Planke et al.^33^ define criteria to identify sill reflections. These reflections must be (1) of high amplitude, (2) have a saucer shape, and (3) terminate abruptly. In the case of the here presented profiles, these scattered reflections have high amplitudes as they are visible well below both the attenuating S-reflection and the top of the oceanic crust and terminate abruptly (Figs. 3, 4, 5). Some of these reflections may have saucer shapes locally, but not all of them. They are associated with outcropping volcanoes in Profiles A and B (Figs. 3, 4). In Profile C, they occur in an area where the top of the oceanic crust has a mounded shape, which may correspond to buried volcanoes (Fig. 5). Complex systems of scattered sills underneath volcanic or hydrothermal mounds have been recognized in the South China Sea based on high-resolution 2D and 3D seismic data^34^. Therefore, we interpret the areas of scattered, high-amplitude reflections in our profiles as zones of complex intrusions associated with surficial or buried volcanic edifices, further consistent with an oceanic basement (Figs. 3, 4, 5).

Apart from the implications of the underlying crust, the presence of volcanic craters implies the widespread occurrence of explosive volcanism along the Red Sea Rift in depths of about 1000 to 1700 m below sea level (mbsl) (at hydrostatic pressures of ~ 10 to 17 MPa). While volcanism at this water depth typically occurs effusively, explosive eruptions of mid-ocean ridge basalts at great depths may be more common than generally assumed^35^. Evidence for phreatomagmatic eruptions in great water depths have been reported from Axial Seamount (~ 1500 mbsl^36^), Gorda Ridge in the NE-Pacific (> 3000 mbsl^37^), the Mid-Atlantic Ridge south of the Azores (500–1750 mbsl^38^) and even the ultra-slow spreading Gakkel Ridge (~ 4000 mbsfl^39^). However, in most cases, only the eruption products were found and the eruption vents were not located nor imaged. Only a few sources show possible deep marine explosion craters near Cap Verde Islands and at the SW Indian Ridge, in water depths of well over 3000 mbsl^40,41^. For explosive eruptions to occur and form crater structures in such great water depths, a high fraction of volatiles that exsolve at high pressure, CO_2_ in particular, dissolved in the magma as well as a rapid depressurization of the underlying magma are required^31^. High magmatic volatiles are unlikely as the composition of the Red Sea lavas represents normal to enriched mid-ocean ridge basalts, with H2O and CO2 values of < 1% (van der Zwan et al.^17^). The fact that the craters occur within the sediment-covered ITZs implies a potentially important role of this cover on the explosivity of the magmas, likely contributing volatiles to an eruption (see also Augustin et al.^4^). The easier removal of the semi-consolidated cover may contribute to quick depressurization.

Alternatively, the volcanic craters can be explained by a piston-like fall of the caldera floor occurring during the withdrawal of magma from an underlying magma chamber, as shown, e.g., at two submarine volcanoes along the Kermadec Arc (Brothers and Rumble II West) where summit depressions have been interpreted as the result of caldera collapse formed by prolonged, episodic, effusive magma withdrawal^42^. At the summit of Loihi Seamount in the Pacific, layered volcaniclastic deposits up to 11 m have been observed along the caldera-forming faults and interpreted as a result of explosive hydrovolcanic activity during caldera collapse^43^, indicating that explosive eruptions in deep water may accompany caldera collapses. This process is unlikely for the craters with an elevated ejection rim but would be possible for craters where an elevated rim is absent^4^.

The lava flow north of the crater at Kebrit Deep (Fig. 6) indicates that the volcanism during crater formation might be associated with explosive to effusive transition^44^, or lava flows associated with or following caldera collapse. Given the broad occurrence of crater structures within sediment-covered parts of the Red Sea (Fig. 1), our study implies that volcanism is widespread along the sediment-covered parts of the Red Sea Rift. Sampling of the crater structures along the Red Sea is required to clarify the eruptive style that led to crater formation.

In conclusion, our study provides compelling evidence for the continuous presence of oceanic crust from the southern Red Sea Rift at least until Mabahiss Deep in the Northern Red Sea, including the sediment-covered Inter-Trough Zones (ITZs). Seismic profiles reveal volcanic structures rising from the oceanic basement and piercing through the evaporite and sediment cover. The widespread occurrence of crater-like structures in the ITZs and seismic evidence of rising volcanic basement indicate that volcanism commonly occurs underneath the sediment-covered regions of the Red Sea Rift. We explain the craters either as a result of explosive deep-sea volcanism at depths of > 1000 m or as collapse structures due to magma withdrawal. These findings support continuous seafloor spreading along large parts of the Red Sea, challenging previous models of disconnected oceanic basins in the central Red Sea.

## Methods

### Multichannel seismics

We present digital multichannel seismic reflection data crossing two ITZs of the Red Sea. They were collected on RV Pelagia during cruise 64PE-445 as part of the SALTAX project^32^. As a seismic source, a Delta Sparker system with a dominant frequency of ~ 300 Hz was used. Seismic energy was recorded using a Microeel solid-state streamer with 24 channels and a length of 100 m (for details, see Augustin et al.^32^). Data processing was carried out using VISTA software and comprised trace-editing, simple frequency filtering (50–2000 Hz), normal moveout correction (1500 m/s), common mid-point stacking, finite-difference post-stack migration, as well as top-muting and white noise removal. Interpretation of the seismic data was carried out using the KingdomSuite software of IHS.

### Bathymetry

Bathymetry and backscatter data were derived from multibeam echosounder data of expeditions Poseidon P408 (Schmidt et al.^45^), Pelagia 64PE-350/351(Schmidt et al.^46^), 64PE-445 (Augustin et al.^32^) and Caladan Oceanic expedition PD20RS02 with RV Pressure Drop. RV Poseidon was equipped with a hull-mounted ELAC NauticSeabeam 3050 echo sounder. RV Pelagia operates a Kongsberg Maritime EM302 multibeam system for water depths down to 3 km. RV Pressure Drop has a full ocean depth Kongsberg Maritime AS EM124 multibeam system. Multibeam bathymetry was processed in QPS Qimera by applying a Medium Spline Filter and manual removal of outliers. Backscatter Mosaic was created in FMGT using automatic processing and the application of a 3-Sigma filter.

## Data Availability

All seismic data collected during expedition 64PE-445 are accessible at Zenodo data repository (https://doi.org/10.5281/zenodo.13739386). All shown bathymetric and backscatter data are available at Pangaea data repository (https://doi.org/10.1594/PANGAEA.860374, https://doi.org/10.1594/PANGAEA.912178) and NOAA bathymetry database (https://www.ngdc.noaa.gov/ships/pressure_drop/PD20RS02_mb.html).
